# Disruption of TWIST1-RELA binding by mutation and competitive inhibition to validate the TWIST1 WR domain as a therapeutic target

**DOI:** 10.1186/s12885-017-3169-9

**Published:** 2017-03-10

**Authors:** Cai M. Roberts, Sophia A. Shahin, Joana Loeza, Thanh H. Dellinger, John C. Williams, Carlotta A. Glackin

**Affiliations:** 1 City of Hope, 1500 E Duarte Rd, Duarte, CA 91010 USA; 2 California State University, 5151 State University Drive, Los Angeles, CA 90032 USA; 3 Present address: Yale University School of Medicine, 333 Cedar Street, New Haven, CT 06520 USA; 4 Present address: University of California, San Francisco, 505 Parnassus Ave, San Francisco, CA 94143 USA

**Keywords:** TWIST1, RELA, WR domain, Protein-protein interactions, Protein degradation

## Abstract

**Background:**

Most cancer deaths result from tumor cells that have metastasized beyond their tissue of origin, or have developed drug resistance. Across many cancer types, patients with advanced stage disease would benefit from a novel therapy preventing or reversing these changes. To this end, we have investigated the unique WR domain of the transcription factor TWIST1, which has been shown to play a role in driving metastasis and drug resistance.

**Methods:**

In this study, we identified evolutionarily well-conserved residues within the TWIST1 WR domain and used alanine substitution to determine their role in WR domain-mediated protein binding. Co-immunoprecipitation was used to assay binding affinity between TWIST1 and the NFκB subunit p65 (RELA). Biological activity of this complex was assayed using a dual luciferase assay system in which firefly luciferase was driven by the interleukin-8 (IL-8) promoter, which is upregulated by the TWIST1-RELA complex. Finally, in order to inhibit the TWIST1-RELA interaction, we created a fusion protein comprising GFP and the WR domain. Cell fractionation and proteasome inhibition experiments were utilized to elucidate the mechanism of action of the GFP-WR fusion.

**Results:**

We found that the central residues of the WR domain (W190, R191, E193) were important for TWIST1 binding to RELA, and for increased activation of the IL-8 promoter. We also found that the C-terminal 245 residues of RELA are important for TWIST1 binding and IL-8 promoter activation. Finally, we found the GFP-WR fusion protein antagonized TWIST1-RELA binding and downstream signaling. Co-expression of GFP-WR with TWIST1 and RELA led to proteasomal degradation of TWIST1, which could be inhibited by MG132 treatment.

**Conclusions:**

These data provide evidence that mutation or inhibition of the WR domain reduces TWIST1 activity, and may represent a potential therapeutic modality.

**Electronic supplementary material:**

The online version of this article (doi:10.1186/s12885-017-3169-9) contains supplementary material, which is available to authorized users.

## Background

The majority of cancer deaths are the result of tumor cells metastasizing beyond their original niche [1]. Disseminated disease is difficult to resect and may be genetically different to the primary tumor [2]. Moreover, acquisition of drug resistance further complicates effective therapeutic approaches. In ovarian cancer in particular, late stage at discovery and drug resistance are major challenges [3, 4], resulting in five year survival rates of approximately 25% [3, 5]. Thus, in ovarian and other cancers, a novel therapeutic strategy capable of addressing both metastasis and drug resistance is urgently needed.

**Fig. 1:**
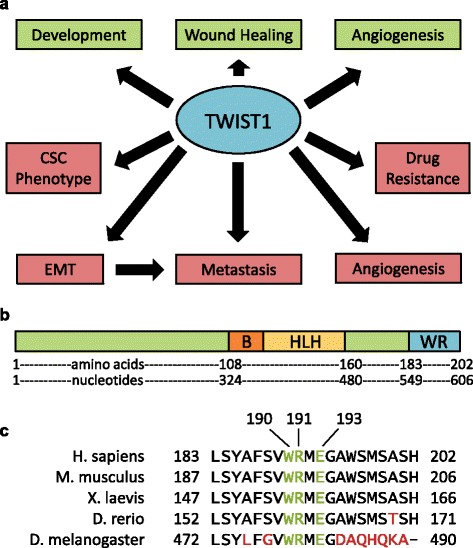
TWIST1 is a highly conserved bHLH class transcription factor with multiple functions. **a** TWIST1 functions in normal development and in small populations of adult stem cells, where it assists in wound healing. When reactivated in cancers, TWIST1 activates a transcriptional and protein binding program giving rise to EMT, and thus to metastases. Many studies have also linked re-expression of TWIST1 to the acquisition of drug resistance and an increase in stemness. Functions in normal tissue are shown in *green*; in cancer, in *red*. **b** Human TWIST1 protein is 202 amino acids in length, with the N-terminal half of the protein being largely disodered The C-terminal half consists of the basic DNA binding domain (*orange*), helix-loop-helix dimerization domain (yellow), and the Twist box or WR domain (*blue*), which has been shown to be a transactivation domain. **c** The WR domain is especially well conserved throughout evolution, with 100% identity between human, mouse, and frog. The central residues appearing in *green* are present in all organisms listed, including *Drosophila*, and for this reason, residues were selected for mutation

A promising target for such an approach is the transcription factor TWIST1. TWIST1 expression and activity is essential in early development but is not retained in adults. However, many cancers reactivate TWIST1 expression [6–8]. In both the developmental and cancer contexts, TWIST1 drives epithelial to mesenchymal transition (EMT), in which cells alter their phenotype, including elongated morphology and expression of cell surface proteins, to facilitate migration and invasion [7]. Enhanced cellular motility in turn gives rise to mesodermal tissues in embryogenesis and to metastases in cancer [7, 8]. Furthermore, TWIST1 has been implicated in number of pro-progression phenotypes in cancers, including angiogenesis [9], increased cancer cell stemness [10–13], and cell survival signaling [14, 15] (Fig. 1a).

TWIST1 has well-characterized transcription factor activity; its dimerization partners and binding site within target promoters have been elucidated previously [16, 17]. Recently, more studies have focused on the Twist box or WR domain, comprised of the C-terminal twenty amino acids of the protein (Fig. 1b). The *TWIST1* gene is well conserved evolutionarily, but this is especially true for the WR domain; 100% homology is preserved from human to Xenopus (Fig. 1c). We have previously shown that the WR domain mediates a binding interaction between TWIST1 and the NF-κB subunit RELA, and that this interaction leads to transcriptional upregulation of the inflammatory cytokine interleukin 8 (IL-8) in a manner independent of TWIST1-DNA binding [18]. Furthermore, Piccinin et al. demonstrated a binding interaction between the WR domain and the C-terminus of the tumor suppressor p53, which led to p53 degradation [19]. Recently, it was revealed that the WR domain can also bind to the WR domain of a nearby TWIST1-E47 heterodimer, thereby creating higher order complexes required for proper transcriptional regulation of target genes [17]. This finding may explain the finding that altered TWIST1-mediated transcription of Hoxa9 was responsible for the inability of prostate cancer cells expressing WR-truncated alleles of *TWIST1* to metastasize in an in vivo model system [20].

Given its importance in mediating not only protein-protein interactions, but also the DNA binding activity of TWIST1, we hypothesize that the WR domain is a potential target to block TWIST1 functions associated with cancer. To test this hypothesis, we sought specific residues mediating the interaction with RELA and tested mutants lacking these residues using our previously validated model system [18]. We further demonstrate that a WR domain mimetic can abrogate TWIST1 activity in vitro, providing further evidence that blocking this interaction and inhibiting TWIST1 expression could be an effective cancer therapeutic strategy.

## Methods

### Cell culture

HEK-293 cells were grown in McCoy’s 5A medium supplemented with 10% fetal bovine serum (FBS) and 1% penicillin/streptomycin (P/S). Ovcar4 cells were grown in RPMI medium with 10% FBS and 1% P/S. All cells were maintained at 37 °C and 90% humidity in a tissue culture incubator with 5% CO_2_ atmosphere. Cells were passaged every 2–4 days as they became confluent, using 0.25% trypsin. Where indicated, cells were transfected using 5 μL per well Lipofectamine 2000 (Life Technologies, Carlsbad, CA) in a total of 2 mL per well of OptiMEM low serum medium (Life Technologies). Cycloheximide (CHX) was obtained from Sigma Aldrich (St. Louis, MO) and used at a dose of 20 μg/ml. For CHX studies, cells were transfected using XtremeGene 9, also from Sigma Aldrich. For proteasome inhibition studies, MG132 was added to HEK-293 cells in normal medium four hours after transfection and left on overnight. A dose of 5 μM was used for fractionated western studies and 1 μM was used for luciferase assays.

### Site directed mutagenesis

The cloning of *TWIST1* into the pcDNA4-MycHis vector has been described previously [18]. The wild type *RELA* gene was also cloned into pcDNA4-MycHis, including a stop codon at the C-terminus to prevent translation of the Myc-His tag. *TWIST1* retained the tag. Amino acid substitution and truncation mutations were introduced using the QuikChange II site directed mutagenesis kit (Agilent, Santa Clara, CA) according to the manufacturer’s instructions and following their recommendations for primer design. Silent mutations were introduced in tandem with the desired mutations in order to create or eliminate restriction sites to facilitate screening for mutants. All mutations were confirmed by Sanger sequencing by the City of Hope Integrative Genomics Core.

### GFP fusion protein

In order to create a competitive inhibitor for TWIST1-RELA binding, the WR domain from TWIST1 was fused to eGFP. Briefly, PCR was used to amplify the final 63 nucleotides of the *TWIST1* gene (including stop codon) and add 5’ XhoI and 3’ BamHI restriction sites. The PCR fragment and the pEGFP-C3 vector were subjected to XhoI-BamHI double digest (New England BioLabs, Ipswich, MA) and the two fragments ligated together. GFP lacking the WR domain was used as a control, and includes 21 residues at the C-terminus encoded by the multiple cloning site of the vector. As a result, the molecular weights of the two GFP proteins are indistinguishable on a western blot. To achieve equal expression of GFP-WR compared to unmodified GFP, it was necessary to transfect cells with three-fold more GFP-WR plasmid versus GFP. A one to one ratio was sufficient for CoIP illustrated in Fig. 4C. For all GFP-WR experiments, 4x refers to GFP-WR only, 3x to a 3:1 ratio of GFP-WR to GFP, 2x to equal amounts of both, 1x to a 1:3 ratio of GFP-WR to GFP, and 0 to GFP only.

### Co-Immunoprecipitation

HEK-293 cells were plated at 500,000 cells per well, in 2 mL normal medium, in a 6 well plate and allowed to adhere. The next day, medium was replaced with OptiMEM low serum medium (Life Technologies). Cells were transfected with various alleles of *TWIST1*, *RELA*, and *GFP* using Lipofectamine 2000 (Life Technologies). The following day, cells were detached using trypsin, washed with PBS, and pelleted. Cell pellets were lysed in RIPA buffer, and protein concentration was determined by BCA Protein Assay (Thermo Fisher, Waltham, MA). 50–100 μg total protein (equal between conditions) was pre-cleared by incubating with 1 μg normal rabbit IgG (Santa Cruz Biotechnology, Dallas, TX) and 20-30 μL Protein A/G Agarose beads (Santa Cruz Biotechnology, sc-2003) on a rocker at 4 °C for 1 h. Water was added to equalize volumes across conditions. Beads were centrifuged for 1 min at 3,000 rpm, and equal volumes of supernatant from each condition were transferred to new tubes, and incubated with 1 μg rabbit anti-RELA (Santa Cruz Biotechnology sc-109) or rabbit anti-GFP (Santa Cruz Biotechnology, sc-8334) antibodies on a rocker at 4 °C. After 1 h, 20–30 μL (equal between conditions) Protein A/G Agarose beads were added to each tube, and tubes were returned to the rocker at 4 °C overnight. The following day, unbound protein was removed and beads were washed five times with 1 mL PBS. Beads were boiled in 20 μL 2x loading dye to release bound protein. Equal masses of input and equal volumes of immunoprecipitated protein were used for western blotting.

### Cell fractionation

HEK-293 cells were plated and transfected as described for co-immunoprecipitation above. The following day, cells were detatched using trypsin, washed with PBS, and pelleted. Pellets were resuspended in 100 μL hypotonic buffer (10 mM HEPES, 10 mM KCl, 0.1 mM EDTA, 1 mM Na_3_VO_4_, 1.25 mM NaF, 0.4% IGEPAL, 0.5 mM DTT) in the presence of protease inhibitor (Thermo Fisher, Waltham, MA). Cells were left on ice 15 min to swell, and then lysed by addition of NP-40 to a final concentration of 0.1%. Nuclei were separated from cytoplasmic lysate by centrifugation (3000 rpm, 10 min, 4 °C) and washed once in hypotonic buffer without NP-40. Nuclei were then resuspended in 50 μL high salt buffer (20 mM HEPES, 400 mM NaCl, 1 mM EDTA, 10% glycerol, 1 mM Na_3_VO_4_, 1.25 mM NaF, 0.5 mM DTT) plus protease inhibitor. Vials were shaken for 2 h at 250 rpm at 4 °C, and then centrifuged (5 min, 14,800 rpm, 4 °C). NaCl concentration was adjusted to 137 mM by addition of water prior to western blotting.

### Cycloheximide study

HEK-293 cells were plated at 150,000 or 250,000 per well in 12 well plates and allowed to adhere overnight. The following day, cells were transfected as described for the above procedures. On the third day, non-treated cells were harvested and cycloheximide was added to the remaining wells. Remaining treated cells were harvested at the indicated time points and used for western blotting.

### Western Blotting

Protein was run on 10% resolving polyacrylamide gels and transferred to PVDF membrane. Membranes were rinsed with PBS and blocked in 5–10% milk, 1 h at room temperature or overnight at 4 °C. Membranes were then incubated with mouse primary antibody in milk with Tween-20 (Ab Buffer) for 1 h at room temperature or overnight at 4 °C, and washed in PBS with 0.1% Tween-20 (PBST). Membranes were then incubated with anti-mouse secondary antibody in Ab Buffer for 1 h at room temperature, followed by an additional five PBST washes. Primary antibodies were: TWIST1, TWIST 2c1a (Santa Cruz Biotechnology sc-81417) 1:250-1:500; for RELA, NF-κB p65 F-6 (Santa Cruz Biotechnology sc-8008) 1:250-1:500; for GFP, GFP B-2 (Santa Cruz Biotechnology sc-9996) 1:1000; for actin, Sigma Aldrich A1978 or 2066. Secondary antibodies were HRP conjugated anti-mouse and anti-rabbit. Protein was detected using Blue Devil Film (Genesee) and ECL Plus (Thermo Fisher) or digital imaging. Quantitation of digital images was performed using the accompanying software from Syngene (Frederick, MD) or Carestream MI (Woodbridge, CT).

### Luciferase assay

Ovcar3 and Ovcar4 cells were plated at 50,000 or 75,000 cells per well, in 500 μL RPMI, in a 24 well plate and allowed to adhere overnight. Ovcar4 cells were used for all luciferase assays except for that shown in Additional file 1: Figure S1. The following day, cells were switched to OptiMEM medium and transfected using Lipofectamine 2000 at 2 μL per well. Plasmids were: *TWIST1* in pcDNA4, *RELA* in pcDNA4, Renilla luciferase, and firefly luciferase (*FFluc*) in pGL3. *FFluc* was under the control of the IL-8 promoter; construction of this vector has been described previously [18]. Empty pGL3 lacking a promoter was used as a negative control for FFluc expression. Each condition was tested in triplicate. The day after transfection, luciferase expression was quantified using the Dual Luciferase Assay kit (Promega, Madison, WI) according to the manufacturer instructions.

### Confocal microscopy

HEK-293 cells were plated at 500,000 per well in glass bottom 35 mm cell culture dishes, and the next day were transfected with TWIST1, RELA, and GFP or GFP-WR as described above. After a further 24 h, cells were rinsed with PBS and stained for 15 min with DAPI. DAPI was then replaced with PBS. Images were captured using a Zeiss LSM700 Confocal Microscope and ZEN 2012 microscopy software (Zeiss AG, Oberkochen, Germany).

### Data analysis and statistics

Western blots were quantified using GeneTools software. Data were graphed and analyzed in Microsoft Excel and GraphPad Prism 6, respectively. Luciferase assays were analyzed using one-way ANOVA with correction for multiple comparisons. For assay testing RELA mutants, all conditions were compared to all others. For assays testing TWIST1 mutants and GFP-WR inhibitor, positive control condition was compared to all others. Positive control conditions are indicated in each relevant figure. Cell counts were averages of four counts, and prior testing has demonstrated that the count is accurate to within 13%. All error bars represent standard deviation. *, *p* < .05; **, *p* < .01; ***, *p* < .001; ****, *p* < .0001 throughout.

## Results

### Single amino acid changes in the WR domain disrupt TWIST1-RELA binding

**Fig. 2:**
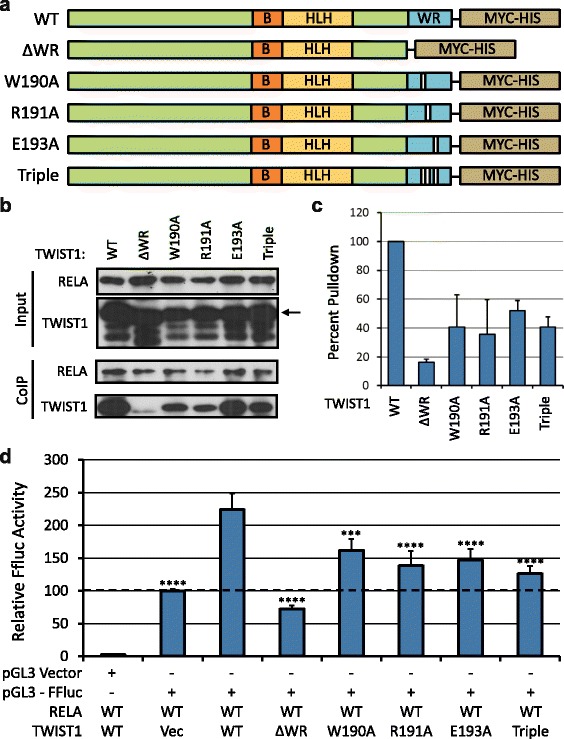
Mutation of the WR domain abrogates TWIST1 interaction with RELA. **a** Schematic representation of TWIST1 alleles used. Triple mutant contains W190A, R191A, and E193A mutations. **b** Co-IP reveals that single amino acid substitutions in the WR domain affect TWIST1-RELA binding, with the triple mutant producing a greater reduction in binding. **c** Quantitation of duplicate CoIP western blots. TWIST1 mutations lead to 50–60% reduction in RELA binding on average. Graphed is the ratio of TWIST1 to RELA, each normalized to its input for each condition. **d** Dual luciferase assay demonstrates that IL-8 promoter driven luciferase activity, a surrogate for IL-8 activation by the TWIST1-RELA complex, is influenced by TWIST1 mutation. As seen in the CoIP, single amino acid substitutions reduce FFluc expression by about 50% with respect to RELA alone, with the triple mutant producing a greater reduction. Graph represents firefly luciferase expression normalized to renilla luciferase for each condition. Error bars represent standard deviations of biological triplicate experiments. WT TWIST1 condition was used as the basis for statistical comparisons. pGL3 lacking the IL-8 promoter was used as a negative control. ***, *p* < .001; ****, *p* < .0001

Site-directed mutagenesis was used to generate mutations in the WR domain of *TWIST1*. On the basis of their high evolutionary conservation (Fig. 1c), we selected W190, R191, and E193 for mutation to alanine (W190A, R191A, E193A alleles, respectively). The ∆WR allele, in which all twenty amino acids of the WR domain have been deleted, was created previously as described elsewhere [18]. Mutants were screened by restriction digestion and confirmed by sequencing (data not shown). All alleles are shown schematically in Fig. 2a. In order to determine the contribution of individual amino acids in the WR domain to TWIST1-RELA binding, we transiently expressed RELA and all TWIST1 alleles in HEK293 cells and performed co-immunoprecipitation (CoIP). Following RELA pulldown, western blotting showed that as demonstrated previously, truncation of the entire WR domain reduced TWIST1 co-precipitation to basal levels. W190A, R191A, and E193A mutations reduced TWIST1 co-precipitation by 50-60%. A triple mutant with W190A, R191A, and E193A mutations also reduced RELA binding by 60%, with less variability (Fig. 2b-c).

### Ability of mutant TWIST1 to drive expression of IL-8 is reduced

We have previously established that formation of a TWIST1-RELA complex upregulates IL-8 expression by 2-2.5 fold over RELA alone, and that prevention of binding by truncating TWIST1 returns IL-8 expression to basal levels [18]. In order to determine the effect of W190A, R191A, and E193A mutations on IL-8 promoter activity, we performed a dual luciferase assay in which firefly luciferase (FFluc) was under the control of the IL-8 promoter. As expected, exogenous expression of RELA in Ovcar4 cells gave rise to a basal level of IL-8 driven FFluc, which was increased by co-expression of, and thus binding with, TWIST1 (Fig. 2d). Mirroring the phenotypes seen in our CoIP experiments above, W190A, R191A, and E193A mutations reduced expression of FFluc by 50%, and the triple mutant reduced FFluc expression a further 10–20% compared to the single point mutants (Fig. 2d). Similar results were obtained using the cell line Ovcar3 (Additional file 1: Figure S1), but a better range of IL-8 promoter induction was achieved in Ovcar4, and this line was selected for all subsequent functional assays.

### RELA C-terminus is required for TWIST1 binding

**Fig. 3:**
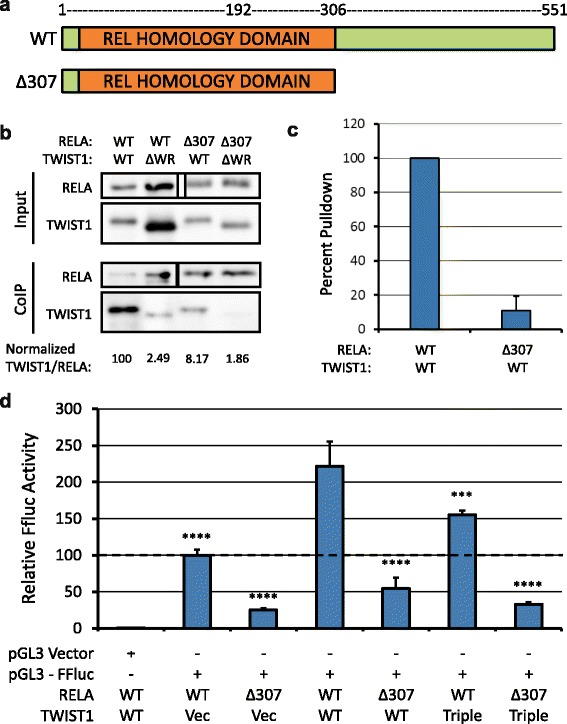
Truncation of RELA reveals TWIST1 binding domain is also required for IL-8 regulatory activity. **a** Schematic representation of RELA alleles. **b** CoIP shows that expression of truncation mutants of either TWIST1 or RELA prevents most binding between TWIST1 and RELA. Co-expression of both truncation mutants further reduces binding, validating the truncated domains as required binding sites for their counterpart proteins. RELA ∆307 bands have been shown separate from WT due to difference in electrophoretic mobility on account of reduced size. **c** Quantitation of duplicate western blots following CoIP of TWIST1 with indicated RELA alleles. ∆307 mutation reduced protein binding by 90% on average. Graphed is the ratio of TWIST1 to RELA, each normalized to their respective inputs. **d** Dual luciferase assay reveals that while the Δ307 allele of RELA reduces TWIST1-mediated upregulation of IL-8 when compared to WT RELA, the same trend is seen in the absence of TWIST1. This suggests that the C-terminal portion of RELA is required not only for TWIST1 binding, but also for proper transcriptional activity. Graph represents firefly luciferase expression normalized to renilla luciferase for each condition. Error bars represent standard deviations of biological triplicate experiments. WT TWIST1 + WT RELA condition was used as the basis for statistical comparisons. pGL3 lacking the IL-8 promoter was used as a negative control. ***, *p* < .001; ****, *p* < .0001

While we have shown that the TWIST1 C-terminus is required for complex formation with RELA, the required residues of RELA remained unknown. In order to locate this site, we created a truncation mutant of RELA, ∆307 (Fig. 3a). Site directed mutagenesis was employed to insert a stop codon directly following the coding sequence for the REL homology domain, a well-conserved domain that has been structurally characterized [21]. CoIP of RELA revealed that truncation of RELA reduced co-precipitation of TWIST1 by approximately 90% (Fig. 3b-c). Truncating both proteins resulted in a greater loss of binding; under these conditions, only 1.86% of wild type levels of TWIST1 was detectable following CoIP (Fig. 3b).

### RELA C-terminus is required for IL-8 activation, independent of TWIST1 mutation status

In order to verify that loss of binding between RELA ∆307 and TWIST1 impacted IL-8 expression, we again utilized a dual luciferase assay. As expected, RELA truncation was able to reduce FFluc expression (Fig. 3d). However, this phenotype was independent of TWIST1; in the absence of TWIST1, RELA ∆307 produced only 30% of wild-type IL-8 promoter activity. TWIST1 expression upregulated IL-8-driven FFluc approximately two-fold, regardless of RELA status. As seen previously, co-expression of triple mutant TWIST1 with RELA led to an intermediate phenotype, for both WT and ∆307 alleles of RELA (Fig. 3d). Thus, we conclude that the domains required for both IL-8 transactivation and complexing with TWIST1 are contained within the relatively uncharacterized C-terminus of RELA.

### Creation of a GFP-WR domain fusion protein

**Fig. 4:**
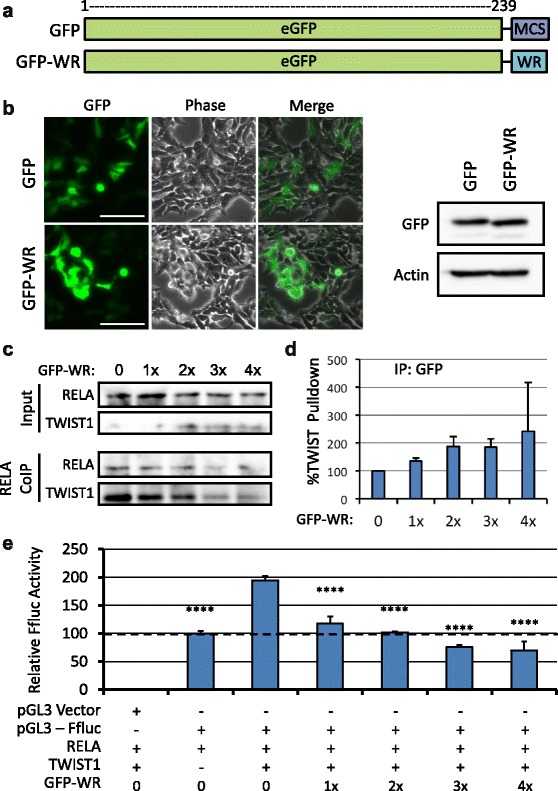
Competitive inhibition of TWIST1 WR domain binding. **a** Schematic representation of GFP alleles used. GFP contains 23 amino acids encoded by the multiple cloning site of the vector at its C-terminus. GFP-WR contains the first two such amino acids (Leu-Glu encoded by XhoI restriction site), followed by the 20 amino acids of the WR domain. Thus, the two alleles have indistinguishable molecular weights. **b** Left, fluorescent microscopy shows that GFP and GFP-WR are expressed at similar levels and in similar patterns in HEK-293 cells. Scale bar, 100 μm. Right, Western blot confirms equal GFP and GFP-WR expression. **c** CoIP with RELA pulldown reveals that in the presence of increasing GFP-WR expression, TWIST1-RELA binding is reduced in a dose-dependent manner. **d** CoIP with GFP pulldown reveals that increasing GFP-WR dose results in more TWIST1 co-precipitated with GFP. Graph represents ratio of TWIST1 to GFP, normalized to their respective inputs. Error bars, standard deviation of duplicate experiments. **e** Dual luciferase assay demonstrates that as seen in the RELA CoIP, there is a dose dependent drop in IL-8 driven luciferase expression with increasing dose of GFP-WR inhibitor. Graph represents firefly luciferase expression normalized to renilla luciferase for each condition. Error bars represent standard deviation of biological triplicate experiments. GFP without GFP-WR condition was used as the basis for statistical comparisons. pGL3 lacking the IL-8 promoter was used as a negative control. ****, *p* < .0001

Given the demonstrated role for the WR domain in RELA binding, as well as in the transcription factor activity of TWIST1 [17, 20], we propose that this domain is an attractive target for therapeutic intervention. To test whether the WR domain could act as a competitive inhibitor of TWIST1-RELA binding, the WR domain was fused to GFP in the pEGFP-C3 vector (Fig. 4a). Empty pEGFP-C3 encodes GFP followed by 21 residues encoded by the multiple cloning site. We therefore used this vector as a negative control, since its protein product would be of the same size as GFP-WR (Fig. 4a). Both forms of GFP could be expressed to similar degrees in HEK293 cells (Fig. 4b).

### GFP-WR fusion protein reduces TWIST1-RELA binding and IL-8 activation

To determine the effect of GFP-WR on TWIST1-RELA binding, we performed CoIP analyses. Total GFP expression in transfected cells was held constant across all conditions by supplementing GFP-WR with control GFP. RELA pulldown revealed that levels of TWIST1 co-precipitated were reduced in a dose dependent fashion with increasing GFP-WR expression (Fig. 4c). GFP pulldown revealed that TWIST1 was co-precipitated in a dose-dependent manner with increasing GFP-WR expression (Fig. 4d). These findings suggest that GFP-WR is interacting with TWIST1 via WR-WR binding, an interaction illustrated by recent studies of higher order TWIST1 complexes [17]. In order to determine whether GFP-WR-mediated inhibition of TWIST1-RELA binding impacted downstream signaling, we again employed a dual luciferase assay to quantify IL-8 promoter activity. As expected, GFP-WR expression led to a dose-dependent reduction in FFluc expression (Fig. 4e). Thus, the TWIST1-driven IL-8 pathway can be inhibited by direct competition using the WR domain.

### GFP-WR fusion protein leads to TWIST1 degradation

**Fig. 5:**
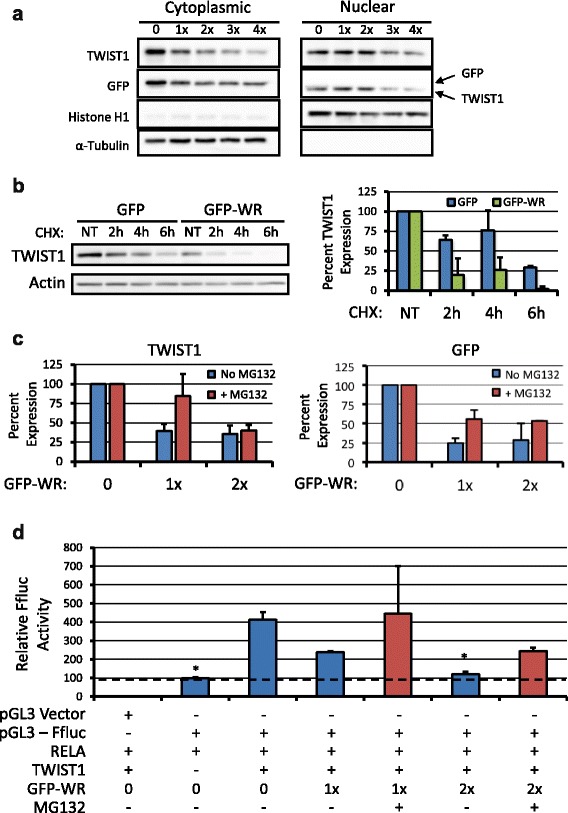
Mechanism of GFP-WR action. **a** Fractionation experiments reveal an overall decrease in TWIST1 and GFP protein expression in the cytoplasm as the level of GFP-WR co-expressed in cells increases. TWIST levels also decrease in the nucleus, but GFP-WR is not expressed in the nuclear fraction. This suggests that GFP-WR expression may lead to TWIST1 degradation. Histone H1 and alpha tubulin were used as nuclear and cytoplasmic markers, respectively. **b** Cycloheximide (CHX) treatment of cells co-transfected with TWIST1 and either GFP or GFP-WR. Left, representative western blot demonstrates more rapid turnover of TWIST1 in the presence of GFP-WR than GFP. Right, quantitation of duplicate experiments. **c** TWIST1 and GFP levels in the cytoplasmic fraction show a 2-fold increase upon MG132 treatment at 1x dose of GFP-WR (biological duplicate experiments, each condition normalized to its 0 GFP-WR control). **d** Dual luciferase assay demonstrates that MG132 treatment increases IL-8 driven FFluc expression. Graph represents firefly luciferase expression normalized to renilla luciferase for each condition. Error bars represent standard deviations of biological triplicate experiments. GFP without GFP-WR or MG132 was used as the basis for statistical comparisons. pGL3 lacking the IL-8 promoter was used as a negative control. Error bars, standard deviation. *, *p* < .05

As GFP-WR was primarily expressed in the cytoplasm of transfected cells (Additional file 1: Figure S2), we hypothesized that GFP-WR was sequestering TWIST1 in the cytoplasm. In order to test this hypothesis, we isolated cytoplasmic and nuclear cell fractions and analyzed the levels of TWIST1 found in each. Western blot of fractionated cells showed that in both cytoplasmic and nuclear fractions, the protein levels of TWIST1 and GFP decreased as the proportion of GFP-WR transfected was increased (Fig. 5a). This suggested that rather than sequestration, the interactions between these proteins may lead to their degradation, as seen previously following altered binding of TWIST1 to partner proteins [19, 22]. In order to test this hypothesis, we transfected HEK-293 cells with TWIST1, RELA, and either GFP or GFP-WR and after 24 h, treated with cycloheximide (CHX) to prevent further protein production. TWIST1 was degraded more quickly in cells expressing GFP-WR than in those expressing GFP (Fig. 5b), suggesting that GFP-WR leads to TWIST1 turnover. To determine if this process was dependent on proteasomal activity, we transefcted HEK-293 cells and treated them with the proteasome inhibitor MG132 overnight. Western blots show that MG132 was able to increase the levels of TWIST1 and GFP by up to two fold in the cytoplasmic fraction of these cells (Fig. 5c). Finally, in order to determine the effect of proteasome inhibition on IL-8 promoter activity, a dual luciferase assay was once again employed. Treatment with MG132 following GFP-WR expression increased IL-8 promoter activity two fold, correlating with increased TWIST1 expression observed following MG132 treatment (Fig. 5d).

## Discussion

We and others have shown that the TWIST1 WR domain is important for TWIST1 protein binding and transcription factor activities, and here we have analyzed further the specific interaction between TWIST1 and RELA. We demonstrated previously that the WR domain was required for the formation of a complex between these two proteins, but that TWIST1-DNA binding was dispensable [18]. We further showed that the production of IL-8 was reduced by loss of binding as a result of deleting the WR domain [18]. In the present study, we identified three highly conserved residues within the WR domain and mutated each to alanine in order to ascertain their role in TWIST1 activity. We observed that all three mutations led to a 50% reduction in TWIST1-RELA co-precipitation and downstream IL-8 promoter activity; the triple mutant further reduced RELA binding and IL-8 promoter activity. These findings suggest that the central region of the WR domain (W190, R191, E193) is important for protein-protein interactions involving TWIST1. This function may explain their evolutionary sequence conservation.

It is important to note that the data presented here cannot preclude the existence of additional or intermediary protein members of the TWIST1-RELA complex, although their overexpression in HEK-293 cells in the absence of other exogenous cofactors and previous work on these two proteins suggests that a direct binding interaction is likely [23].

Further studies, including structural biology approaches, will be necessary to fully elucidate the TWIST1-RELA binding interaction. No crystal structure for full length TWIST1 presently exists. However, a computational model predicts a helical structure for much of the WR domain and also suggests an interface that binds to p53 [19]. The R191 residue in particular was responsible for disrupting p53 post-translational modifications, leading to p53 degradation [19]. We have shown here that the WR domain interacts with a RELA transactivation domain downstream of the REL homology domain, which also has yet to be structurally characterized. Other groups have shown also that the WR-domain of TWIST1 binds to Sox10 and Runx3 [24, 25], and additional binding partners may yet be identified. Further studies are needed to recognize structural motifs that may predict TWIST1-binding sites on additional cellular proteins.

Having shown that the bHLH domain of TWIST1 was not required for IL-8 regulation [18], we hypothesized that separation of function would be possible, and that we could independently study the DNA binding and protein binding functions of TWIST1. However, Gajula et al. showed that TWIST1 lacking the WR domain was unable to promote metastasis in an in vivo model of prostate cancer. Specifically, they found that TWIST1-mediated regulation of Hoxa9 at the transcriptional level was responsible for the phenotype they observed [20]. A possible explanation for this finding is that TWIST1-responsive promoters can contain tandem E-box sequences. Both E-boxes are bound by TWIST1 heterodimers, which then interact via their WR domains to form a transient tetramer [17]. Thus, whether directly bound to DNA or bound to protein cofactors, there is now strong evidence that WR domain interactions lie at the heart of many TWIST1 signaling processes.

Targeting of the WR domain offers a potential therapeutic approach to simultaneously disrupt protein binding, transcription factor activities, and rate of recycling of the TWIST1 protein. To test this hypothesis, a GFP fusion protein including the WR domain was created and used to inhibit normal TWIST1-RELA complex formation and IL-8 promoter regulation. The GFP-WR fusion protein successfully reduced TWIST1 activity, and led to TWIST1 degradation in a dose dependent manner. The finding that TWIST1 was co-precipitated with GFP-WR suggests that these proteins are interacting via their WR domains, blocking WR domain binding to other partner proteins.

Importantly, TWIST1 inhibition via blocking of binding and subsequent degradation has a natural analogue, supporting its efficacy: TWIST1 is known to be sequestered by HLH inhibitor of DNA binding (Id) family members 2 and 4, preventing its binding to other partners [26, 27]. Moreover, mutations in TWIST1 found in Saethre-Chotzen Syndrome that prevent its dimerization and nuclear translocation have been shown to lead to degradation of the protein [22].

Future work will focus on further characterization of TWIST1 turnover and GFP-WR mechanism. It is possible that binding between the WR domains of TWIST1 and GFP-WR alone is sufficient to lead to TWIST1 degradation, but it is also possible that the GFP component of the fusion protein contributes to TWIST1 inhibition. For instance, the size of the protein may block other TWIST1 binding partners from binding, or prevent post-translational modifications of TWIST1 required for its stability or activity. To assess the efficacy of the WR domain alone, we have created a WR domain peptide fused to a nona-arginine cell penetrating leader sequence, and will test its ability to inhibit TWIST1 binding and activity. Use of this peptide design is supported by the efficacy of a similar peptide mimic of BRCA1-IRIS, which led to degradation of IRIS and reversal of its pro-drug resistance effects [28].

Future work will also focus on additional therapeutics against TWIST1. We have already demonstrated the efficacy of siRNA against TWIST1 delivered using multiple nanoparticle platforms. Dendrimers carrying siRNA against TWIST1 reduced migration in vitro and homed to tumors in a xenograft model of triple negative breast cancer in mice [29]. In addition, mesoporous silica particles carrying chemically modified siRNA reduced tumor size in an in vivo melanoma model via reduction of angiogenesis [30] and reversed cisplatin resistance in an ovarian cancer model, leading to reduction of tumor growth in mice [31]. We have also shown that cells exhibited reduced Akt signaling and in vivo survival in response to cisplatin treatment when TWIST1 expression was reduced by shRNA [15].

TWIST1 is an attractive target for novel therapies: it is rarely expressed in adult tissues, reducing the chance of off-site effects [32], and it plays a role in multiple cancer processes correlated with poor outcome, such as metastatic spread, angiogenesis, resistance to apoptosis, drug resistance pathways, and cancer cell stemness (Fig. 1a) [7, 9, 11, 14, 33, 34]. Further development of multiple approaches to TWIST1 targeting is warranted, as patients at the highest risk, and who therefore tend to have the fewest therapeutic options, may be in a position to benefit most from TWIST1-targeted treatment.

## Conclusions

We have shown here that the formation of a TWIST1-RELA complex is partially dependent on the well conserved W190, R191, and E193 residues of the TWIST1 WR domain, and that mutations at these positions reduce binding and downstream IL-8 promoter activation. These effects may be the result of lower binding affinity due to changes to the binding interface or to destabilization of the WR domain fold. We have further showed that the C-terminus of RELA is involved both in TWIST1 binding and IL-8 promoter activation. Finally, we have demonstrated a competitive inhibitor of the TWIST1 WR domain has therapeutic potential, leading to the degradation of TWIST1 protein. Further investigations of WR-mediated binding interactions and development of TWIST1-targeted therapies may be of great value to patients suffering from advanced, drug resistant carcinomas.

## Additional file

Additional file 1:
**Figure S1.** TWIST1 mutation decreases IL-8 promoter activity in Ovcar3 cells. Replicating the experiment shown for Ovcar4 in Fig. 2 shows a similar trend in the Ovcar3 cell line. As Ovcar3 showed a smaller degree of induction with TWIST1 expression, Ovcar4 was used as the cell line of choice for remaining functional assays. **Figure S2.** Confocal images of GFP allele localization. GFP lacking the WR domain is visible throughout the cell, including the nucleus, as indicated by DAPI staining. GFP-WR signal appears distinct from DAPI-positive nuclei. (PDF 456 kb)

## Abbreviations

CHX: Cycloheximide
CoIP: Co-immunoprecipitation
CSC: Cancer stem cell
EMT: Epithelial to mesenchymal transition
FBS: Fetal bovine serum
FFluc: Firefly luciferase
GFP: Green fluorescent protein
GFP-WR: GFP-WR domain fusion protein
Id: Inhibitor of DNA binding
IL-8: Interleukin 8
PBS: Phosphate buffered saline
PBST: PBS with 0.1% Tween-20
