# Supplementary material for: Disruption of TWIST1-RELA binding by mutation and competitive inhibition to validate the TWIST1 WR domain as a therapeutic target
Source: BMC Cancer. 2017 Mar 10;17:184. doi: 10.1186/s12885-017-3169-9 (PMC5345230; doi:10.1186/s12885-017-3169-9)
Supplement: Additional file 1: — Figure S1. TWIST1 mutation decreases IL-8 promoter activity in Ovcar3 cells. Replicating the experiment shown for Ovcar4 in Fig. 2 shows a similar trend in the Ovcar3 cell line. As Ovcar3 showed a smaller degree of induction with TWIST1 expression, Ovcar4 was used as the cell line of choice for remaining functional assays. Figure S2. Confocal images of GFP allele localization. GFP lacking the WR domain is visible throughout the cell, including the nucleus, as indicated by DAPI staining. GFP-WR signal appears distinct from DAPI-positive nuclei. (PDF 456 kb) [file 12885_2017_3169_MOESM1_ESM.pdf]

Supplement to:

Disruption of TWIST1-RELA binding by mutation and competitive inhibition to validate the TWIST1 WR domain as a therapeutic target

Cai M. Roberts, Sophia A. Shahin, Joana Loeza, Thanh H. Dellinger, John C. Williams, and Carlotta A. Glackin

Figure S1

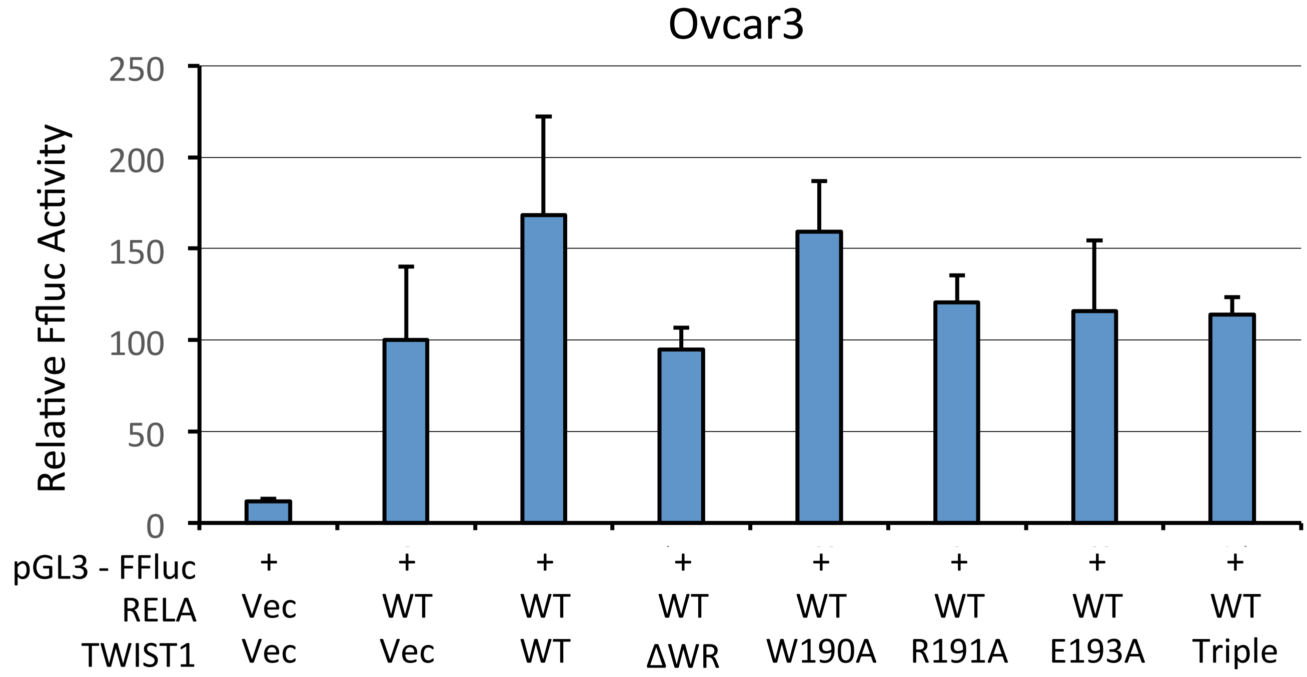

Figure S1. TWIST1 mutation decreases IL-8 promoter activity in Ovcar3 cells. Replicating the experiment shown for Ovcar4 in Figure 2 shows a similar trend in the Ovcar3 cell line. As Ovcar3 showed a smaller degree of induction with TWIST1 expression, Ovcar4 was used as the cell line of choice for remaining functional assays.

Figure S2

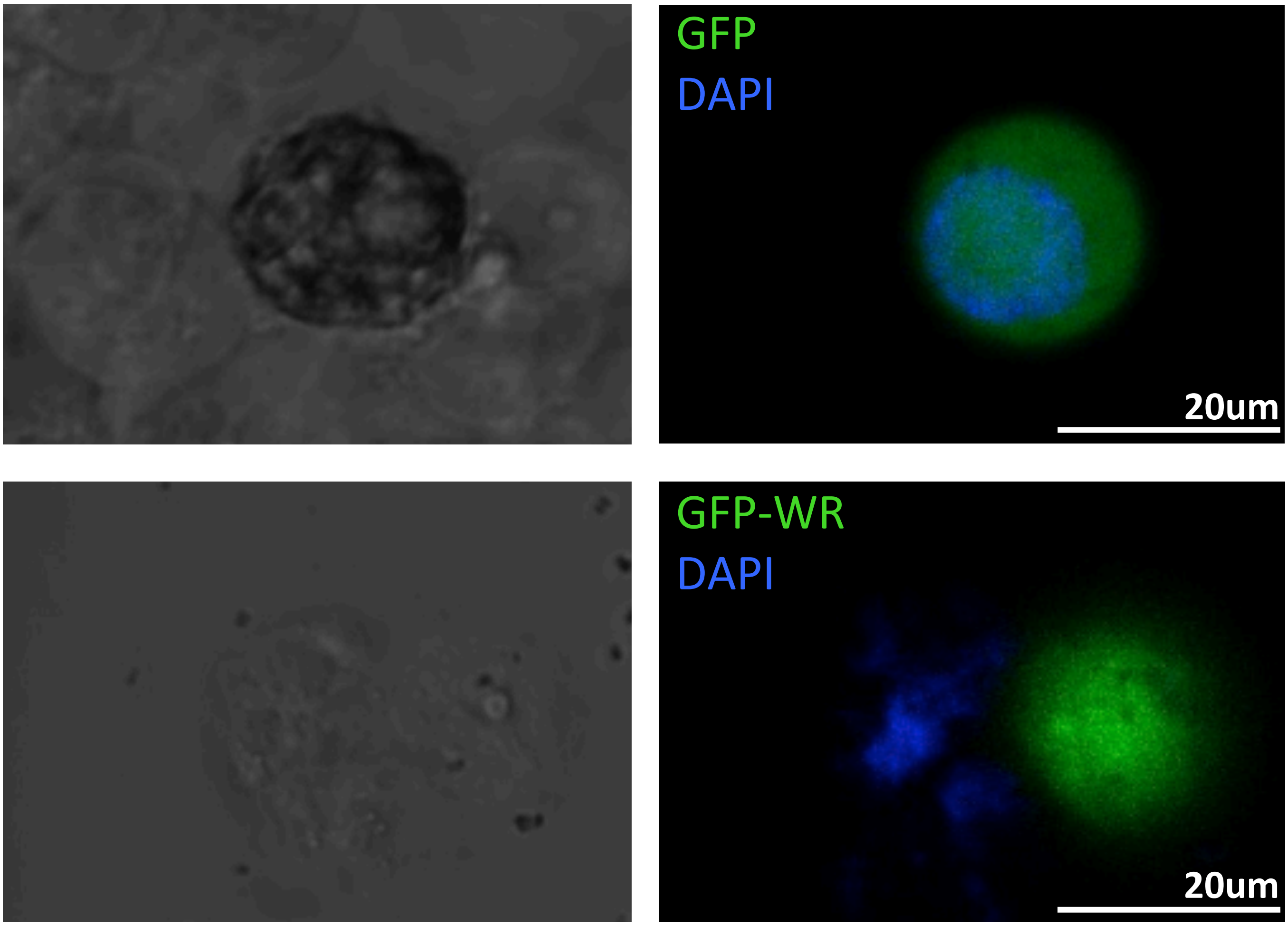

Figure S2. Confocal images of GFP allele localization. GFP lacking the WR domain is visible throughout the cell, including the nucleus, as indicated by DAPI staining. GFP-WR signal appears distinct from DAPI-positive nuclei.
